# Is there coordination of leaf and fine root traits at local scales? A test in temperate forest swamps

**DOI:** 10.1002/ece3.5421

**Published:** 2019-07-09

**Authors:** Yu‐Kun Hu, Xu Pan, Xue‐Jun Yang, Guo‐Fang Liu, Xu‐Yan Liu, Yao‐Bin Song, Man‐Yin Zhang, Li‐Juan Cui, Ming Dong

**Affiliations:** ^1^ Institute of Wetland Research Chinese Academy of Forestry Beijing China; ^2^ Beijing Key Laboratory of Wetland Services and Restoration Beijing China; ^3^ State Key Laboratory of Vegetation and Environmental Change, Institute of Botany Chinese Academy of Sciences Beijing China; ^4^ Key Laboratory of Ecosystem Network Observation and Modeling, Institute of Geographic Sciences and Natural Resources Research Chinese Academy of Sciences Beijing China; ^5^ Key Laboratory of Hangzhou City for Ecosystem Protection and Restoration, College of Life and Environmental Sciences Hangzhou Normal University Hangzhou China

**Keywords:** aboveground‐belowground linkages, fine roots, forest swamps, functional traits, local scales, successional gradient

## Abstract

Examining the coordination of leaf and fine root traits not only aids a better understanding of plant ecological strategies from a whole‐plant perspective, but also helps improve the prediction of belowground properties from aboveground traits. The relationships between leaf and fine root traits have been extensively explored at global and regional scales, but remain unclear at local scales. Here, we measured six pairs of analogous leaf and fine root traits related to resource economy and organ size for coexisting dominant and subordinate vascular plants at three successional stages of temperate forest swamps in Lingfeng National Nature Reserve in the Greater Hinggan Mountains, NE China. Leaf and fine root traits related to resource acquisition (e.g., specific leaf area [SLA], leaf N, leaf P, root water content, and root P) decreased with succession. Overall, we found strong linear relationships between leaf dry matter content (LDMC) and root water content, and between leaf and root C, N, and P concentrations, but only weak correlations were observed between leaf area and root diameter, and between SLA and specific root length (SRL). The strong relationships between LDMC and root water content and between leaf and root C, N, and P held at the early and late stages, but disappeared at the middle stage. Besides, C and P of leaves were significantly correlated with those of roots for woody plants, while strong linkages existed between LDMC and root water content and between leaf N and root N for herbaceous species. These results provided evidence for the existence of strong coordination between leaf and root traits at the local scale. Meanwhile, the leaf–root trait relationships could be modulated by successional stage and growth form, indicating the complexity of coordination of aboveground and belowground traits at the local scale.

## 1 INTRODUCTION

Plant functional traits define ecological strategies and the adaption of plants to environments (Westoby & Wright, 2006). The variation and covariation in functional traits aid to understand the responses of community properties and ecosystem functioning to environmental changes (Díaz & Cabido, 2001; Funk et al., 2017). Among these, an important question is how different suites of functional traits are correlated with each other (Westoby & Wright, 2006; Wright et al., 2004). Focusing on specific organs related to different functions, researchers have found some leading dimensions of trait variation, for example, leaf economics spectrum (Wright et al., 2004), wood economics spectrum (Chave et al., 2009), and root economics spectrum (Roumet et al., 2016). Recently, from the perspective of whole plant, some researchers have examined the trait relationships across organs, for example, whole‐plant economics spectrum (Díaz et al., 2016; Freschet, Cornelissen, Logtestijn, & Aerts, 2010; Silva, Souza, Caliman, Voigt, & Lichston, 2018).

The linkages between leaf and root traits are among the hottest topics in investigating the trait covariation of whole plants (Craine, Lee, Bond, Williams, & Johnson, 2005). On the one hand, both leaves and roots (especially for fine roots) are related to resource acquisition, and their linkages are critical to understanding the ecological strategies of whole plants. On the other hand, fine root traits are far less understood than leaf traits (Ma et al., 2018); thus, exploring leaf–root trait coordination is crucial to predicting belowground traits from aboveground ones. A large number of studies have investigated the leaf–root trait relationships at regional and global scales. For grassland and savannah plant species, Tjoelker, Craine, Wedin, Reich, and Tilman (2005) found a strong concordance in leaf and root N and longevity, but not in specific leaf area (SLA) and specific root length (SRL). Consistent relationships between leaf and root traits, especially chemical traits, were found in semi‐arid and arid ecosystems (Cheng, Chu, Chen, Bai, & Niu, 2016; Liu et al., 2010), alpine ecosystems (Geng, Wang, Jin, Liu, & He, 2014), and temperate forests (Valverde‐Barrantes, Smemo, Blackwood, & Norden, 2015). At larger scales (global), relationships between leaf and root N, and between SLA and SRL have been well quantified (Craine et al., 2005; Kerkhoff, Fagan, Elser, & Enquist, 2006; Valverde‐Barrantes, Freschet, Roumet, & Blackwood, 2017). These findings seem to support the whole‐plant economics spectrum at regional and global scales. Examining the coordination between leaf and root traits at local scale would aid revealing ecological processes within communities, for example, community assembly (Holdaway, Richardson, Dickie, Peltzer, & Coomes, 2011). However, whether root traits are coordinated with leaf traits at local scales remains unclear.

Nonetheless, a large amount of variation in leaf and root traits has been observed in various ecosystems at the local scale (Hu et al., 2015; Liu et al., 2010; Messier, McGill, & Lechowicz, 2010; Wright et al., 2004). The drivers of trait variation are often different across spatial scales; for example, both climatic and soil conditions were important determinants at global and regional scales, while soil water and nutrient availability were the main factors at local scales (Messier et al., 2010). The trends of trait relationships may be contrasting across scales (Messier, McGill, Enquist, & Lechowicz, 2017). Thus, it is vital to examine whether the global‐ or regional‐scale leaf–root relationships will hold at local scales. If there are inconsistent leaf–root trait correlations between local and global or regional scales, it would indicate the scale dependence of these relationships (Messier, McGill, et al., 2017). Furthermore, exploring the coordination of leaf and root traits at local scales is necessary to test the generalization of leaf–root relationships. However, current studies about the local‐scale linkages between leaf and root traits have been few and contradictory. According to Liu et al. (2010), there were strong correlations between leaf N and root N, and between SLA and SRL at the local scale in semi‐arid and arid ecosystems. However, for Mediterranean forests, the coordination of leaf and root morphological traits and resource‐use traits became weaker or disappeared while shifting from a regional scale into a local scale (de la Riva et al., 2016). These studies proved the context dependent of trait covariation, indicating that different ecosystems may be potential drivers of this contradiction.

As the major drivers of plant trait variation at local scales, soil factors have been frequently reported to change predictably along the succession, for example, decreasing soil nutrient availability (Holdaway et al., 2011). Leaf traits vary systematically with the soil chronosequence (Buzzard et al., 2016; Holdaway et al., 2011; Lohbeck et al., 2013; Shipley, Vile, & Garnier, 2006). Shifts in fine root traits during succession have been less investigated; thus, leaf–root trait relationships within and across successional stages are poorly understood. Previous studies showed that environmental factors could constrain the relationships between leaf and root traits (Craine et al., 2005; Geng et al., 2014). For example, Craine et al. (2005) found that factors such as soil freezing and the type of nutrient limitation appeared to determine the global relationships between leaves and roots. Similarly, Geng et al. (2014) found a positive SLA‐SRL relationship in temperate grasslands, but a negative relationship in alpine grasslands. These results highlight the need to explore the trait relationships between leaves and roots in different environments. The environmental gradients along the chronosequence of forest swamps were very steep, which provided an ideal system to test the existence of covariation in leaf and root traits at local scale.

Functional groups, for example, growth forms (herbaceous and woody), photosynthetic pathways (C_3_, C_4_ and CAM), summarized major functional differences among species (Lavorel, McIntyre, Landsberg, & Forbes, 1997). There exist large differences in both leaf and root traits between herbaceous and woody species due to plant strategies. Compared with woody species, herbaceous plants tended to have higher tissue N and P (Kerkhoff et al., 2006), higher SRL, lower root tissue density, and thinner roots (Freschet et al., 2017; Ma et al., 2018). Besides, plant growth forms could shape the strength of leaf–root trait relationships (Kerkhoff et al., 2006; Valverde‐Barrantes et al., 2017).

In this study, we aimed to investigate the coordination of leaf and fine root functional traits at the local scale to test the generalization of the linkages between aboveground and belowground traits. Specifically, we hypothesized that (a) conservative traits would increase and acquisitive traits would decrease with succession, for there was a decrease in soil nutrient along the succession; (b) leaf and root functional traits should be strongly correlated within and across successional stages; (c) there would be strong trait coordination between leaves and roots in both herbaceous and woody species, and the strength should vary between growth forms. To test these hypotheses, we measured six pairs of analogous leaf and fine root traits related to resource economy and organ size of dominant and subordinate plants in a temperate forest swamp along a successional gradient.

## 2 MATERIALS AND METHODS

### 2.1 Study region and sampling

This study was conducted at Lingfeng National Nature Reserve (52°15′–52°31′N, 122°41′–123°26′E) in the Greater Hinggan Mountains, NE China. The climate is typical cold‐temperate in this region, where mean annual temperature is about −5°C, and mean annual precipitation is 500 mm. The main soil types are gelic cambosols, permagelic gleyosols, and orthic spodosols (Editorial Committee of Soil Geography in China, 2014). Coniferous forests, the zonal vegetation in this region, occupy the largest areas. Owing to the Emuer River, there exist large areas of forest swamps, which constitute the second largest vegetation in the Greater Hinggan Mountains (Editorial Committee of Wetland Vegetation in China, 1999). Forest swamps are mainly distributed at riparian zones, ecotones between coniferous forests and lakes, or in the valley (Editorial Committee of Wetland Vegetation in China, 1999).

In this study, we used the method of space‐for‐time substitutions to choose three main types of forest swamps along a successional gradient in a riparian zone. The three stages of chronosequences were found within a short distance (<5 km). The succession of forest swamps was mainly caused by the expansion of *Sphagnum*, which lowered soil pH and nutrient availability (Rydin & Jeglum, 2013). Thus, there was a clear gradient of soil nutrient availability and soil pH for the three forest swamps during succession (Table 1). At the early successional stage, the forest swamp has higher water‐table level, soil nutrient availability, and soil pH with some resource‐acquisitive dominant plants, for example, *Larix*, *Carex* (Table 1). At the middle‐successional stage, there is middle water‐table level, nutrient availability, and soil pH, and the dominant species are *Larix*, *Vaccinium,* and moss (Table 1). At the late stage, the forest swamp is characterized as lower water‐table level, soil nutrient availability, and soil pH, where dominant species consists of *Larix*, *Ledum*, *Vaccinium,* and *Sphagnum* (Table 1; Editorial Committee of Wetland Vegetation in China, 1999).

**Table 1:** The biotic and abiotic properties of forest swamps at the three successional stages

Characteristics	Early stage	Middle stage	Late stage
Dominant species	*Larix gmelini*, *Betula platyphylla*, *Carex schmidtii*, *Deyeuxia angustifolia*	*Larix gmelini*, *Vaccinium uliginosum*, *Vaccinium vitis*‐*idaea*	*Larix gmelini*, *Ledum palustre*, *Vaccinium vitis*‐*idaea*
Species richness	7.9a	5.9b	6.4b
Soil water content (%)	64.8a	41.0ab	26.3b
Soil pH	5.47a	4.70b	4.32c
Soil C:N	16.3a	23.9b	28.2c
Soil N (mg/g)	1.19a	1.02ab	0.25b
Soil P (mg/g)	0.24a	0.17a	0.11a

Note

Values were arithmetic means. Different letters (a, b, and c) indicate significant differences among successional stages at *p* = 0.05.

In each type of forest swamp, we randomly set five plots, and within each plot, one tree quadrat (10 m × 10 m), two shrub quadrats (2 m × 2 m), and five herb quadrats (1 m × 1 m) were used to record the percentage of cover of trees, shrubs, and herbs, respectively. We carried out vegetation survey and sample collection in July 2017. Dominant and subordinate vascular plants of each layer (Table S1), which account for more than 95% of total cover, were chosen to collect leaf and fine root samples. The plant species studied were grouped into two growth forms, that is, woody and herbaceous (Table S1), for shrubs were more similar to trees in many functional traits based on previous studies (Díaz et al., 2016; Valverde‐Barrantes et al., 2017). In each type of forest swamp, three batches of mature and unfolded sun leaves from different individuals were collected for each dominant and subordinate plant species. Meanwhile, three batches of intact roots for each species were carefully excavated at the soil depth of 0–20 cm. For trees and large shrubs, each batch of roots was collected from each individual plant, while for small shrubs and herbs, several individuals were sampled for each batch to ensure enough fine root material for measurement. To obtain exact root samples for specific species, we excavated fine roots based on the aboveground parts of plants. All leaf and fine root samples were stored in separate sealed plastic bags and kept cool within 8 hr before measurement (Pérez‐Harguindeguy et al., 2013).

### 2.2 Leaf and fine root traits

Twelve leaf and fine root traits, related to resource economy and organ size, were used to describe the functional variation of leaves and fine roots (Díaz et al., 2016). Leaf samples were measured for six traits: leaf area, specific leaf area (SLA), leaf dry matter content (LDMC), leaf carbon content (leaf C), leaf nitrogen content (leaf N), and leaf phosphorus content (leaf P). A subsample from each batch of leaf samples were submerged in water overnight (about 12 hr), blotted up water with clean papers, and scanned into images using a photo scanner (Epson Perfection V39; Epson, Japan). Leaf area (cm^2^) was accessed from these images using ImageJ (http://imagej.nih.gov/ij/). After that, each batch of scanned leaf samples was weighed after being oven‐dried at 65°C for 72 hr. The SLA (mm^2^/mg) of each sample was calculated by the ratio of total leaf area to oven‐dry weight, and LDMC (mg/g) was measured by the ratio of leaf oven‐dry weight to water‐saturated weight. Leaf C (mg/g) and leaf N (mg/g) were determined using an elemental analyzer (vario MICRO cube; Elemental, Germany), while leaf P (mg/g) was determined using Inductively Coupled Plasma‐Optical Emission Spectrometry (ICP‐OES prodigy 7; Teledyne Leeman Labs).

All fine root samples were measured for root diameter, specific root length (SRL), root water content, root carbon content (C), root nitrogen content (root N), and root phosphorus content (root P). Fine roots (diameter < 2 mm) were carefully cut from each sample of fresh roots and weighed. These fine roots were then scanned into images using the photo scanner, and total length and averaged diameter of each sample (mm) were accessed from these images using WinRHIZO Pro (Regent Instruments Inc.). Oven‐dry weight of each root sample was obtained after being dried at 60°C for 48 hr. Then, SRL (m/g) was calculated by the ratio of total root length to oven‐dry weight, and root water content (%) was the ratio of root water weight to root oven‐dry weight. Root C (mg/g), root N (mg/g), and root P (mg/g) were determined using the same methods as leaf C, leaf N, and leaf P.

### 2.3 Soil variables

Five soil samples from 0 to 20 cm depth were randomly collected at each plot. Some fresh samples were used to measure soil water content as soon as possible, while the others were air‐dried. About 10 g of fresh soil from each sample was weighed both before and after being oven‐dried at 105°C for 6 hr. Soil water content (%) was calculated as the ratio of soil water mass to oven‐dry weight of each soil sample. All air‐dried soil samples were passed through a 2‐mm sieve before measurement. For soil pH, 5 g subsample of each soil sample was shaken with 12.5 ml demineralized water in glass beaker for 1 min and measured with a pH Meter (PB‐10; Sartorus) after standing for 30 min. For soil carbon (C) content, soil N content, and soil P content, a small amount of each sample which passed through a 0.15‐mm sieve was used. Soil C (mg/g), soil N (mg/g), and soil P (mg/g) contents were determined using the same methods as leaf C, N, and P. Soil C:N was calculated as the ratio of soil C to soil N.

### 2.4 Data analysis

First of all, variance partitioning was used to explore the relative extent of trait variation across three scales nested one into another, that is, within species, across species within successional stages, and across successional stages. To achieve this, we fitted a general linear model to the variance across scales using the restricted maximum likelihood (REML) method. Trait data at individual levels were analyzed with the function *lme* of R package “*nlme*” (Pinheiro, Bates, DebRoy, & Sarkar, 2016). Before the following analysis, leaf area and fine root diameter were log_10_‐transformed to meet the assumptions of normality and equal variance, while other traits were not transformed.

To investigate the effects of successional stage and growth form on leaf and fine root traits, we performed one‐way analysis of variance (ANOVA) and Tukey's multiple comparisons. We also compared species richness, soil water content, soil pH, soil C:N, soil N, and soil P among successional stages using the same analysis. These analyses allowed us to investigate how leaf and root traits vary across successional stages and growth forms. Then, principal component analysis was carried out to assess the coordination of leaf and fine root traits across successional stages and growth forms. Besides, standardized major axis (SMA) regression was specifically used to examine the correlation between analogous leaf and root functional traits across and within successional stages and growth forms. Both principal component analysis and SMA regression allowed us to assess the coordination of leaf and fine root traits at the local scale. We use SMA regression to test how leaf–root trait relationships vary across successional stages and growth forms. Meanwhile, Pearson's correlation analysis was performed to explore the relationships between each pair of leaf and fine root traits. All analyses were conducted using R version 3.2.3 (R Core Team, 2015).

## 3 RESULTS

### 3.1 Trait variation across successional stages, growth forms, and scales

**Figure 1:**
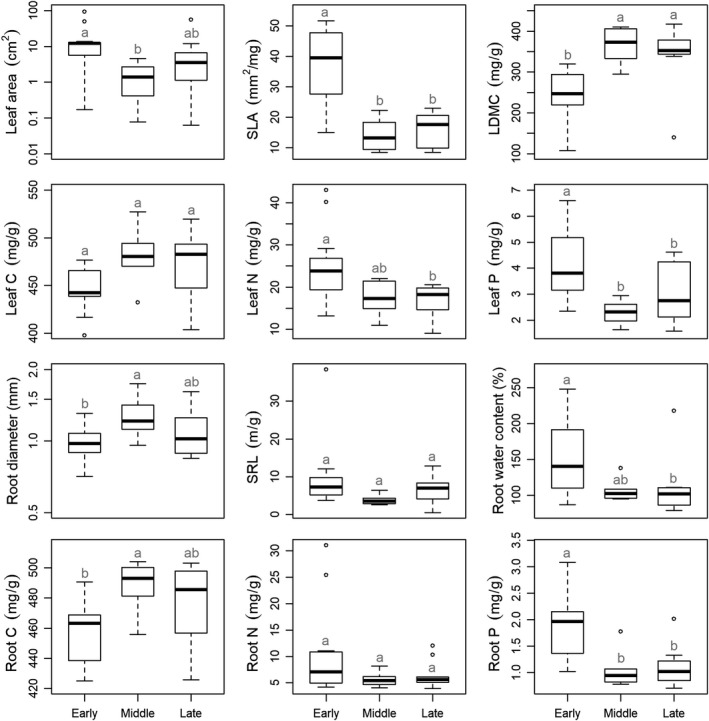
Leaf and fine root traits among the three successional stages (early, middle and late) of forest swamps. Different letters indicate significant differences (*p* < 0.05) in plant traits among successional stages. LDMC, leaf dry matter content; SLA, specific leaf area; SRL, specific root length. Leaf area and root diameter were log_10_‐transformed

Most leaf and fine root traits varied significantly among successional stages (*p* < 0.05), except for leaf C (*F*_2,26_ = 3.185, *p* = 0.058), SRL (*F*_2,26_ = 1.838, *p* = 0.179), and root N (*F*_2,26_ = 1.723, *p* = 0.198; Figure 1). Leaf area (*F*_2,26_ = 3.699, *p* = 0.039) of plants at the early successional stage was significantly larger than that at the middle stage, but similar to that at the late stage, while root diameter (*F*_2,25_ = 3.741, *p* = 0.038) and root C (*F*_2,26_ = 4.255, *p* = 0.025) were the lowest at the early stage (Figure 1). Leaf N (*F*_2,26_ = 4.788, *p* = 0.017) and root water content (*F*_2,26_ = 3.888, *p* = 0.033) were significantly higher at the early stage than that at the late stage, and they were not significantly different between any other two successional stages (Figure 1). SLA (*F*_2,26_ = 15.30, *p* < 0.001), leaf P (*F*_2,26_ = 6.755, *p* = 0.004), and root P (*F*_2,26_ = 9.584, *p* < 0.001) at both middle and late stages were significantly lower than that at the early stage, while there was no significant difference in the three traits between middle and late stages (*p* > 0.05, Figure 1). The LDMC (*F*_2,26_ = 9.417, *p* < 0.001) had the opposite patterns compared with those three traits (Figure 1).

Furthermore, except leaf N, root diameter, and SRL, other leaf and fine root traits differed greatly between woody and herbaceous plants (Figure S1). Leaf area, SLA, leaf P, root water content, root N, and root P of herbaceous plants were significantly larger than that of woody plants (*p* < 0.05), and LDMC, leaf C, and root C of herbaceous species were significantly smaller than that of woody species (*p* < 0.05, Figure S1). Variance component partitioning showed that variation in leaf and root traits differed among the three scales: within species, among species within successional stages, and among successional stages (Figure S2). The largest proportion of total variance in all traits, except for SLA and root P, occurred among species within successional stages, ranging from 49.0% to 88.3% (Figure S2). For SLA and root P, successional stages accounted for the largest proportion of total variance, that is, 56.6% and 35.9%, respectively (Figure S2).

### 3.2 Leaf–fine root trait relationships within and across successional stages

**Figure 2:**
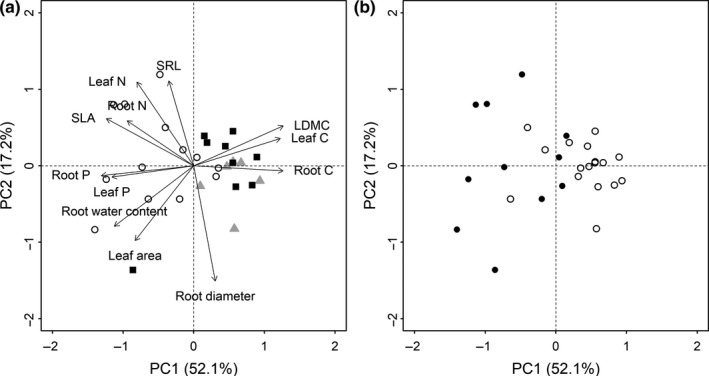
Principal component analysis for all leaf and root traits across three successional stages (a) and plant growth forms (b). (a) symbols: ○, early; 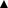, middle; ■, late. (b) symbols: ●, herbaceous; ○, woody. The first axis (PC1) explained 52.1% of total variation, while the second axis (PC2) accounted for 17.2% of total variation

The results of principal component analysis showed that SLA, LDMC, leaf C, leaf N, leaf P, SRL, root water content, root C, root N, and root P concentrated on the first axis, explaining 52.1% of total variation (Figure 2a). The second axis including leaf area, root diameter, and SRL accounted for 17.2% of total variation (Figure 2a). Besides, species at the early successional stage occurred at the left of first PC axis, while species at middle and late stages concentrated at the right of first PC axis (Figure 2a). Meanwhile, herbaceous plants were distinguished from woody plants by clustering at the left of first PC axis (Figure 2b). Leaf economic traits including SLA, LDMC, leaf N, and leaf P were correlated with each other, and root economic traits, that is, SRL, root water content, root C, root N, and root P were also closely related with each other (Table S2).

**Figure 3:**
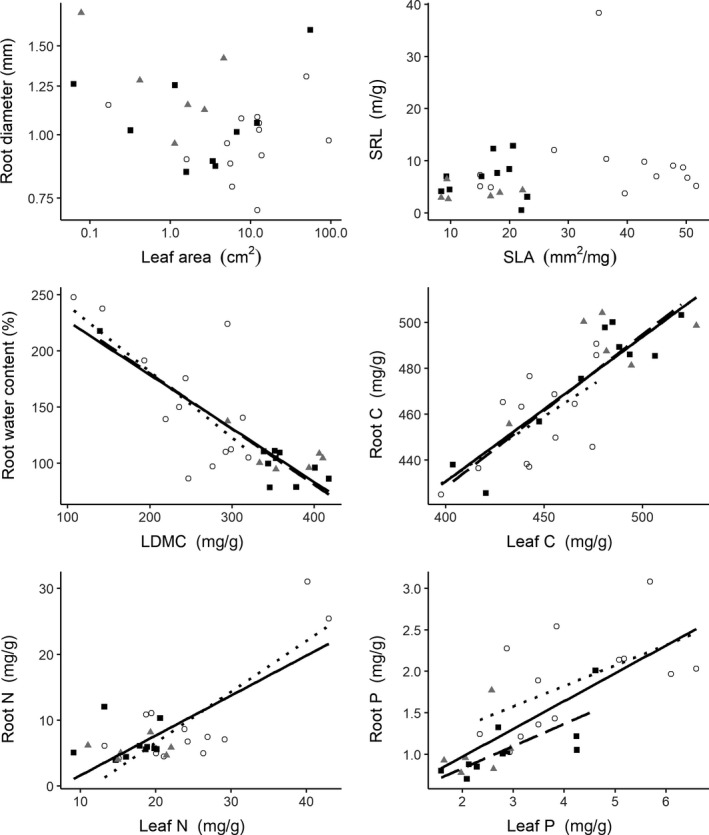
Relationships between leaf and root traits overall and at each successional stage. Symbols: ○, early; 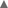, middle; ■, late. Regression lines: dotted line, early; gray line, middle; long dash line, late; solid black lines, overall. Lines were only plotted for those relationships with *p* < 0.05. Leaf area and root diameter were log_10_‐transformed. Full names for the abbreviation can be seen in Figure 1

We found significant correlations between LDMC and root water content (overall, *R* ^2^ = 0.660, *p* < 0.001; early stage, *R* ^2^ = 0.487, *p* = 0.008; late stage, *R* ^2^ = 0.882, *p* < 0.001), between leaf C and root C (overall, *R* ^2^ = 0.682, *p* < 0.001; early stage, *R* ^2^ = 0.427, *p* = 0.015; late stage, *R* ^2^ = 0.840, *p* < 0.001), and between leaf P and root P (overall, *R* ^2^ = 0.534, *p* < 0.001; early stage, *R* ^2^ = 0.321, *p* = 0.043; late stage, *R* ^2^ = 0.566, *p* = 0.012) across successional stages and at early and late stages (Figure 3). Strong positive relationships between leaf N and root N were only found overall (*R* ^2^ = 0.555, *p* < 0.001) and at the early stage (*R* ^2^ = 0.660, *p* < 0.001; Figure 3). However, there was weak linear relationship between leaf area and root diameter (overall, *R* ^2^ = 0.059, *p* = 0.212; early stage, *R* ^2^ = 0.001, *p* = 0.925; middle stage, *R* ^2^ = 0.316, *p* = 0.246; late stage, *R* ^2^ = 0.025, *p* = 0.687; Figure 3). We also observed weak correlations between SLA and SRL (overall, *R* ^2^ = 0.063, *p* = 0.189; early stage, *R* ^2^ < 0.001, *p* = 0.972; middle stage, *R* ^2^ = 0.001, *p* = 0.962; late stage, *R* ^2^ = 0.004, *p* = 0.861; Figure 3).

### 3.3 Leaf–fine root trait relationships between growth forms

Relationships between analogous leaf and fine root traits were different for woody and herbaceous plants (Table 2). We found significant positive correlations between leaf C and root C (*R* ^2^ = 0.304, *p* = 0.022) and between leaf P and root P (*R* ^2^ = 0.851, *p* < 0.001) for woody plants, while strong linkages between LDMC and root water content (*R* ^2^ = 0.687, *p* < 0.001) and between leaf N and root N (*R* ^2^ = 0.709, *p* < 0.001) were observed for herbaceous species (Table 2).

**Table 2:** Results for standardized major axis regression (SMA) between leaf and fine root traits in woody and herbaceous species

Leaf–fine root trait	Woody			Herbaceous		
	*R* ^2^	*p*	*n*	*R* ^2^	*p*	*n*
Leaf area–root diameter	0.170	0.100	17	0.075	0.417	11
SLA‐SRL	0.090	0.241	17	<0.001	0.801	12
LDMC–root water content	0.228	0.052	17	**0.687**	**<0.001**	12
Leaf C–root C	**0.304**	**0.022**	17	0.211	0.132	12
Leaf N–root N	0.161	0.110	17	**0.709**	**<0.001**	12
Leaf P–root P	**0.851**	**<0.001**	17	0.225	0.119	12

Note

Leaf area and root diameter were log_10_‐transformed.

Abbreviations: LDMC, leaf dry matter content; SLA, specific leaf area; SRL, specific root length.

Significant linear relationships (*p* < 0.05) were marked in bold.

## 4 DISCUSSION

In the forest swamp, we found that plants at the early stage tended to have larger leaf area, SLA, leaf N, leaf P, root water content, and root P, but lower LDMC, root diameter, and root C. These supported our first hypothesis, that is, conservative traits would increase and acquisitive traits would decrease with succession. There existed strong correlations between LDMC and root water content, and between leaf and root chemical traits rather than morphological traits across successional stages. These strong correlations occurred at early and late successional stages but not at the middle stage, which partly supported our second hypothesis. Consistent with our third hypothesis, trait coordination of leaves and roots varied between woody and herbaceous plants. All these results above provided evidence for the existences of strong coordination between leaf and root traits at local scale in forest swamps. Meanwhile, the strength of the coordination could be shaped by successional stage and plant growth form.

### 4.1 Leaf and fine root traits varied across successional stages and growth forms

Plant species at the early stage were more resource‐acquisitive, which was consistent with the finding that root N and P decreased and root diameter increased with the succession of a temperate rain forest (Holdaway et al., 2011). The shifts in leaf and root traits during succession can be explained by the changes in soil properties. In this study, the succession of the forest swamp was mainly determined by increasing soil acidity (Rydin & Jeglum, 2013), which caused the decrease in soil nutrient availability (Table 1; Sartoretto, 1991). Meanwhile, there were no significant differences in most leaf and root functional traits between middle and late successional stages, which were probably due to the relatively few differences in soil nutrients between these two stages (Table 1). Soil nutrients and microclimate conditions changed with successional stages (Holdaway et al., 2011; Lohbeck et al., 2013). Thus, changes in plant functional traits during succession depended largely on the shifts in soil and microclimate conditions (Lohbeck et al., 2013). It had important implications for understanding and predicting vegetation change during ecosystem development. The changes in leaf traits with succession had been extensively investigated (Buzzard et al., 2016; Lohbeck et al., 2013; Shipley et al., 2006); however, changes in fine root traits during succession are poorly understood (Holdaway et al., 2011). Our study on both leaf and fine root traits enhanced our understanding of whole‐plant ecological strategies in succession of forest swamps.

The larger root N in herbaceous species than woody ones was consistent with the result of Kerkhoff et al. (2006), who found that herbaceous species had higher leaf and root N than woody species in global seed plants. Leaf N, root diameter, and SRL did not differ significantly between growth forms, which was inconsistent with some previous studies. For example, Freschet et al. (2017) observed that fine roots of herbaceous species were on average finer and had higher SRL than those of woody species, while Ma et al. (2018) found lower root tissue density, thinner roots, larger SRL, and less mycorrhizal colonization in herbaceous plants than in woody species across the globe. There seemed to be much overlap in leaf N, root diameter, and SRL between herbaceous and woody species. We found that herbaceous plants tended to be more resource‐acquisitive than woody species in a forest swamp with a limited number of species.

### 4.2 Effects of successional stage on leaf and root trait relationships

Previous regional‐scale studies have shown consistent positive relationships between leaf and root chemical traits across different environments (Craine et al., 2005; Freschet et al., 2010; Geng et al., 2014; Liu et al., 2010). Our study observed inconsistent correlations between leaf and root chemical traits among successional stages at a local scale. There were two main explanations for these results. First, local environmental conditions may have played important role for the trait variation and covariation of plants. At the middle stage of forest swamps, lower soil pH and nutrient availability could cause strong filtering for the variation of leaves and roots, which were supported by the very restricted range of trait variation (Table S3; Figures 1 and 2a). Previous studies also found that there was an increase of environmental filtering during succession (Buzzard et al., 2016; Caplan, Meiners, Flores‐Moreno, & McCormack, 2019). Second, lowest species richness and diversity in clades (e.g., families) seemed to result in weak leaf–root relationships at the middle stage (Table 1). Correlations between leaf and root chemical traits tended to be weak within limited phylogenetic clades (Valverde‐Barrantes et al., 2017). Overall, our study indicated the environmental dependence of leaf–root trait relationships at local scales.

### 4.3 Effects of growth form on leaf and root trait relationships

As compared with succession stage, growth form modified the strength of leaf–root trait relationships largely. Significant correlations between leaf and root C and P were found for woody plants, while strong linkages between LDMC and root water content and between leaf N and root N were observed for herbaceous species. These results were inconsistent with previous studies, which found strong positive relationships between leaf and root N and P and between SLA and SRL in both woody and herbaceous species (Kerkhoff et al., 2006; Valverde‐Barrantes et al., 2017). The weak leaf–root trait relationships within growth forms might be related to the small sample size (woody, *n* = 17; herbaceous, *n* = 12), because for those nonsignificant leaf–root trait relationships, the power of explanation was relatively big with *R* ^2^ ranging from 0.161 to 0.228 (Table 2). However, these results probably indicated different coordination between leaf and root traits in different growth forms.

### 4.4 Coordination of leaf and fine root traits at local scales

Strong covariation in leaf and root chemical traits at the local scale was consistent with previous global and regional studies (Craine et al., 2005; Kerkhoff et al., 2006; Liu et al., 2010; Tjoelker et al., 2005; Valverde‐Barrantes et al., 2017, 2015). These reflected the strong coordination of leaf and root traits in relation to resource capture and utilization while responses to different availability of soil water and nutrients (Osunkoya, Bayliss, Panetta, & Vivian‐Smith, 2010). Generally, plant species with smaller diameter and high SRL were thought to be more acquisitive use of resources (Liu et al., 2010; Ma et al., 2018). The decoupling between leaf and root morphological traits may be due to the fact that plant nutrient foraging depends largely on mycorrhizal colonization besides absorptive fine roots for nutrient‐limited ecosystems (Valverde‐Barrantes et al., 2017). Our findings suggested that there may be different integration of morphological and chemical properties between above‐ and belowground organs (Geng et al., 2014; Wang, Wang, Zhao, Yu, & He, 2017). Since previous studies found uncertainty in the local‐scale coordination between leaf and fine root traits (Freschet et al., 2010; Silva et al., 2018), the coordination of aboveground and belowground traits seemed to be more complex at the local scale.

Overall, our study provided an empirical evidence of leaf–root linkages at the local scale, but it is uncertain whether this evidence can be generalized in other local scale studies. Therefore, more attention should be paid to other aspects of the aboveground–belowground trait coordination at the local scale. Future studies could focus on (a) the coordination of other leaf and root traits, for example, traits related to mechanical support or transport (Messier, Lechowicz, McGill, Violle, & Enquist, 2017; Reich, 2014), or (b) on the trait coordination in other types of ecosystems (e.g., forests and grasslands), or (c) on not only the relationships between leaf and root traits among species, but also within species (Medeiros, Burns, Nicholson, Rogers, & Valverde‐Barrantes, 2017).

## 5 CONCLUSIONS

In a forest swamp ecosystem, we found strong correlations between LDMC and root water content, and between leaf and root chemical traits, providing evidence that there is coordination of leaf and root traits at the local scale. Our results also indicated that the strength of coordination depended on plant growth forms and successional stages of ecosystems. The inconsistent patterns in the coordination of leaf and root traits at different successional stages might be mainly driven by differences in soil nutrient availability. Moreover, the observed coordination between leaf and root traits at the local scale was weaker but more complex than that at regional and global scales (Silva et al., 2018). This indicated the critical role of scales in influencing the leaf–root trait coordination. Future studies on the coordination of leaf and root traits at different scales will give us more accuracy in predicting belowground traits (in most cases, “hard traits”) from aboveground traits (relatively “soft traits”) and improve our understanding in the complex linkages between above‐ and belowground from local to global scales.

## CONFLICT OF INTEREST

None declared.

## AUTHOR CONTRIBUTIONS

YKH conceived the study; YKH, XP, and XYL collected the data; YKH, XJY, GFL, and YBS analyzed the data and made the figures; YKH, XP, XJY, GFL, XYL, YBS, MYZ, LJC, and MD contributed to writing and editing the manuscript. All authors contributed critically to the drafts and gave final approval for publication.

### DATA ACCESSIBILITY

Data are available from the Dryad Digital Repository (https://doi.org/10.5061/dryad.6cn55fb).
