# Supplementary material for: Is there coordination of leaf and fine root traits at local scales? A test in temperate forest swamps
Source: Ecol Evol. 2019 Jul 9;9(15):8714–23. doi: 10.1002/ece3.5421 (PMC6686282; doi:10.1002/ece3.5421)
Supplement: Supplementary file 1 [file ECE3-9-8714-s001.docx]

**Is there coordination of leaf and fine root traits at local scales? A test in temperate forest swamps**

**Yu-Kun Hu^1,2^, Xu Pan^1,2^, Xue-Jun Yang^3^, Guo-Fang Liu^3^, Xu-Yan Liu^4^, Yao-Bin Song^5^, Man-Yin Zhang^1,2^*, Li-Juan Cui^1,2^*, Ming Dong^5^**

* Corresponding author

Man-Yin Zhang and Li-Juan Cui, Institute of Wetland Research, Chinese Academy of Forestry, Beijing 100091, China

Emails: [cneco@126.com](mailto:cneco@126.com); [lkyclj@126.com](mailto:lkyclj@126.com)

**Supporting Information**

**Table S1. Species abundance of sampled plants at the three successional stages.**

| Plant species | Growth form | Family | Successional stage | | |
| --- | --- | --- | --- | --- | --- |
|  |  |  | Early | Middle | Late |
| *Larix gmelini* | Woody | Pinaceae | 75.00 | 65.00 | 60.00 |
| *Betula platyphylla* | Woody | Betulaceae | 25.00 |  | 3.00 |
| *Carex schmidtii* | Herbaceous | Cyperaceae | 14.50 | 4.06 | 3.74 |
| *Ribes nigrum* | Woody | Saxifragaceae | 9.56 |  |  |
| *Rubus arcticus* | Herbaceous | Rosaceae | 8.96 |  |  |
| *Deyeuxia angustifolia* | Herbaceous | Poaceae | 4.75 |  |  |
| *Saussurea japonica* | Herbaceous | Compositae | 3.20 |  |  |
| *Pyrola dahurica* | Herbaceous | Pyrolaceae | 1.92 |  |  |
| *Thalictrum aquilegifolium* var. *sibiricum* | Herbaceous | Ranunculaceae | 1.88 |  |  |
| *Spiraea salicifolia* | Woody | Rosaceae | 1.24 |  |  |
| *Vicia ramuliflora* | Herbaceous | Leguminosae | 1.08 |  |  |
| *Vicia cracca* | Herbaceous | Leguminosae | 0.92 |  |  |
| *Rosa davurica* | Woody | Rosaceae | 0.88 |  |  |
| *Betula fruticosa* | Woody | Betulaceae |  | 0.76 | 3.84 |
| *Ledum palustre* | Woody | Ericaceae |  | 12.48 | 34.16 |
| *Vaccinium uliginosum* | Woody | Ericaceae |  | 29.16 | 2.54 |
| *Vaccinium vitis*-*idaea* | Woody | Ericaceae |  | 31.44 | 29.40 |
| *Salix hsinganica* | Woody | Salicaceae |  |  | 1.36 |
| *Sanguisorba officinalis* | Herbaceous | Rosaceae |  |  | 0.68 |
|  |  |  |  |  |  |
| All species not sampled |  |  | 5.51 | 4.38 | 0.76 |
|  |  |  |  |  |  |
| Percent total cover sampled |  |  | 96.40 | 97.03 | 99.46 |

Note: the mean percent cover is given for each sampled species at each successional stage. The percentage of total cover sampled at each stage is also given.

**Table S2. Pearson’s correlation analysis between each pair of plant traits for all species in this study.**

|  |  | Leaf | | | | | | | | Fine root | | | | | |
| --- | --- | --- | --- | --- | --- | --- | --- | --- | --- | --- | --- | --- | --- | --- | --- |
|  |  | | Leaf area | SLA | LDMC | Leaf C | Leaf N | Leaf P | Root diameter | | SRL | Root water content | Root C | Root N | Root P |
| Leaf | Leaf area | | - | 29 | 29 | 29 | 29 | 29 | 28 | | 29 | 29 | 29 | 29 | 29 |
|  | SLA | | **0.26*** | - | 29 | 29 | 29 | 29 | 28 | | 29 | 29 | 29 | 29 | 29 |
|  | LDMC | | **- 0.62**** | **- 0.68**** | - | 29 | 29 | 29 | 28 | | 29 | 29 | 29 | 29 | 29 |
|  | Leaf C | | **- 0.55**** | **- 0.64**** | **0.68**** | - | 29 | 29 | 28 | | 29 | 29 | 29 | 29 | 29 |
|  | Leaf N | | <0.01 | **0.73**** | **- 0.33**** | - 0.05 | - | 29 | 28 | | 29 | 29 | 29 | 29 | 29 |
|  | Leaf P | | **0.49**** | **0.75**** | **- 0.70**** | **- 0.67**** | **0.40*** | - | 28 | | 29 | 29 | 29 | 29 | 29 |
| Root | Root diameter | | <0.01 | **- 0.25*** | 0.05 | **- 0.24*** | **- 0.29**** | - 0.15 | - | | 28 | 28 | 28 | 28 | 28 |
|  | SRL | | 0.06 | **0.22*** | - 0.09 | - 0.17 | 0.09 | 0.09 | **- 0.48**** | | **-** | 29 | 29 | 29 | 29 |
|  | Root water content | | **0.50**** | **0.36**** | **- 0.70**** | **- 0.53**** | - 0.05 | **0.54**** | 0.10 | | - 0.04 | - | 29 | 29 | 29 |
|  | Root C | | **- 0.46**** | **- 0.57**** | **0.57**** | **0.82**** | 0.05 | **- 0.57**** | **- 0.21*** | | **- 0.32**** | **- 0.59**** | **-** | 29 | 29 |
|  | Root N | | 0.13 | **0.52**** | **- 0.43**** | **- 0.35**** | **0.51**** | 0.26 | - 0.11 | | 0.11 | **0.51**** | **- 0.45**** | **-** | 29 |
|  | Root P | | **0.38*** | **0.76**** | **- 0.76**** | **- 0.67**** | **0.41*** | **0.73**** | - 0.13 | | 0.13 | **0.81**** | **- 0.71**** | **0.59**** | **-** |

Note: SLA, specific leaf area; LDMC, leaf dry matter content; SRL, specific root length. * *p* < 0.05, ** *p* < 0.01.

Table S3. Ranges (max-min) of plant trait values at different successional stages of forest swamps

| Trait | Early | Middle | Late |
| --- | --- | --- | --- |
| Leaf area (cm^2^) | 94.1 | 4.6 | 55.6 |
| SLA (mm^2^ mg^-1^) | 36.7 | 13.8 | 14.6 |
| LDMC (mg g^-1^) | 212.6 | 115.8 | 278.4 |
| Leaf C (mg g^-1^) | 78.9 | 95.0 | 115.8 |
| Leaf N (mg g^-1^) | 29.8 | 11.1 | 11.5 |
| Leaf P (mg g^-1^) | 4.2 | 1.3 | 3.0 |
| Root diameter (mm) | 0.59 | 0.78 | 0.77 |
| SRL (m g^-1^) | 34.6 | 3.8 | 12.3 |
| Root water content (%) | 161.1 | 42.6 | 139.0 |
| Root C (mg g^-1^) | 65.8 | 48.5 | 77.7 |
| Root N (mg g^-1^) | 26.8 | 4.1 | 8.1 |
| Root P (mg g^-1^) | 2.0 | 1.0 | 1.3 |

Note: full names for the abbreviation are the same as Table S2.


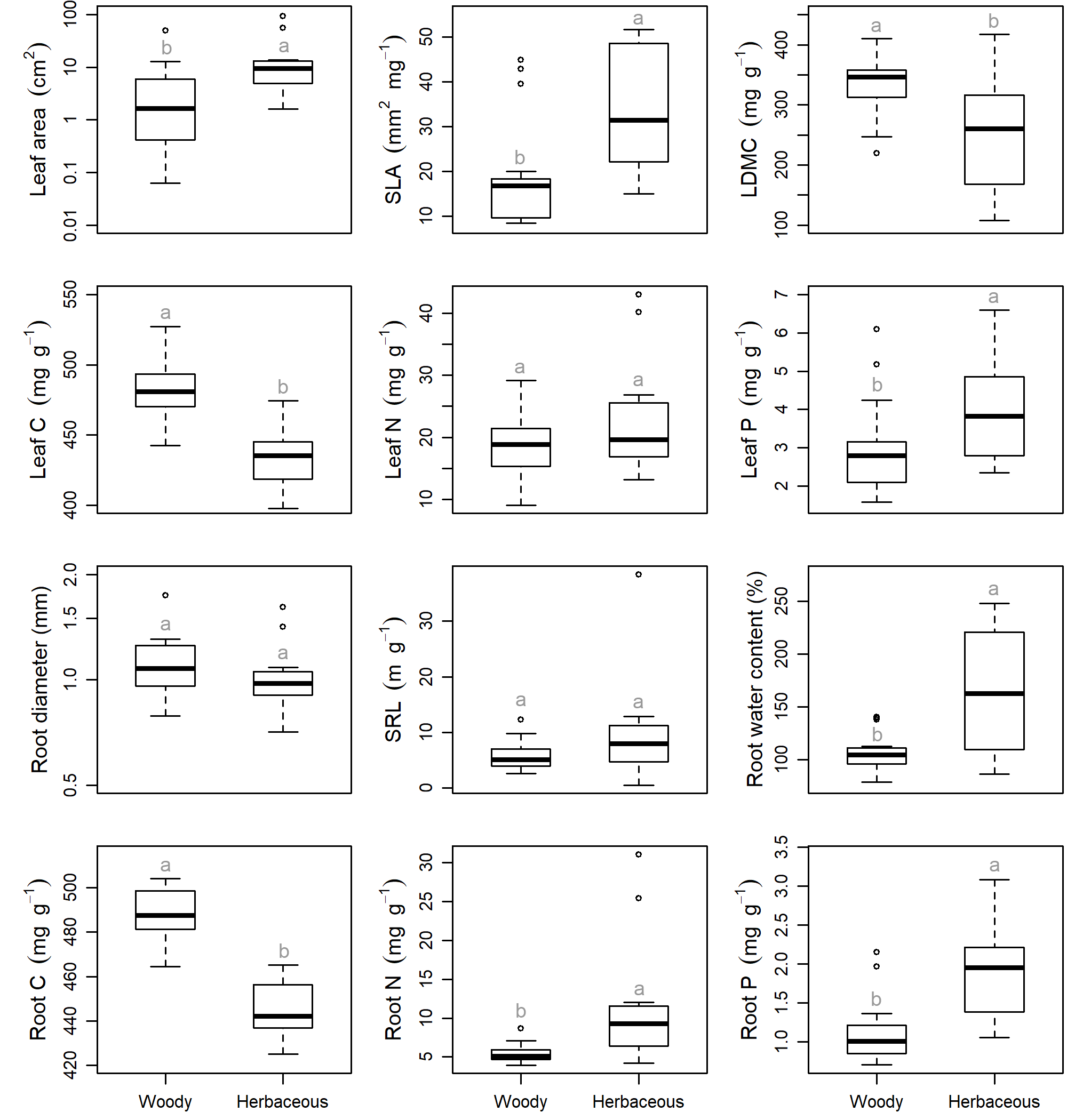


**Figure S1. Leaf and fine root traits in plants of different growth forms (woody vs herbaceous).** Different letters indicate significant differences (*p* < 0.05) in traits between woody and herbaceous plants. SLA, specific leaf area; LDMC, leaf dry matter content; SRL, specific root length. Leaf area and root diameter were log_10_-transformed.


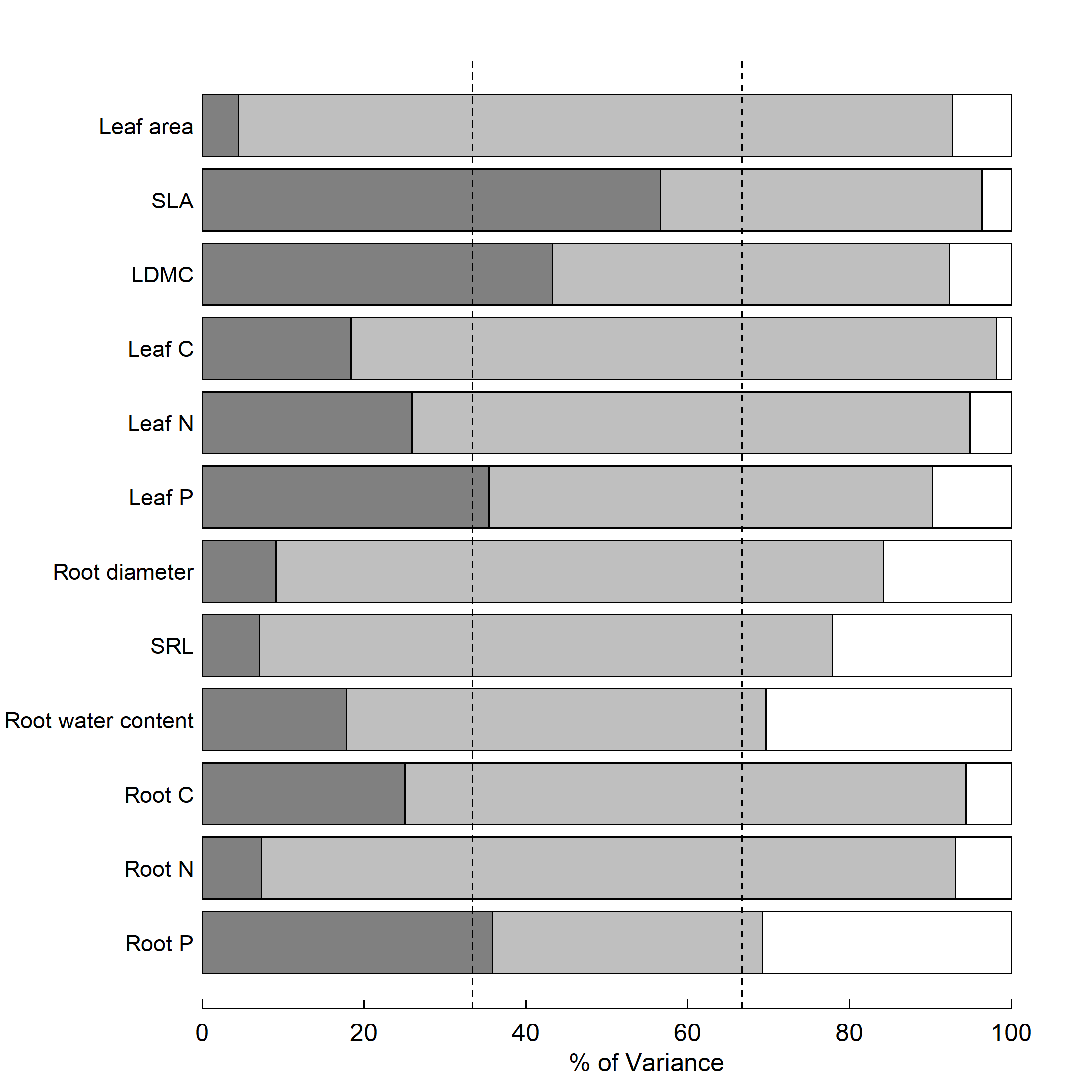


**Figure S2. The relative extent of variation in leaf and root traits across the three scales.** ■, among successional stages; ■, among species within successional stages; ■, within species. The 33.3% and 66.6% thresholds are given by dash lines. Full names for the abbreviation can be seen in Figure S1.
